# Editorial: Advancements in hematopoietic stem cell proliferation and self-renewal maintenance

**DOI:** 10.3389/fcell.2025.1607145

**Published:** 2025-07-07

**Authors:** Pawan Kumar Raghav, Gurudutta Gangenahalli, Takahiko Hara

**Affiliations:** ^1^ Immunogenetics and Transplantation Laboratory, Department of Surgery, University of California San Francisco, San Francisco, CA, United States; ^2^ Netaji Subhas University of Technology Delhi, Delhi, India; ^3^ Tokyo Metropolitan Institute of Medical Science Tokyo, Tokyo, Japan

**Keywords:** Hematopoietic stem cells (HSCS), Self-renewing populations (SRPs), Bone marrow-pathology, Long-term culture-initiating cells (LTC-ics), Hematopoietic stem and progenitor cells (HSPCs), Induced pluripotent stem cells (iPSCs), Self-renewal, Proliferation

Hematopoietic stem cells (HSCs) are the foundation of blood and immune system regeneration, with their self-renewing populations (SRPs) serving as a primary reservoir within the bone marrow (Rodriguez-Fraticelli et al., 2018). These cells replenish precursor populations and support the hierarchical development of myeloid, lymphoid, and erythroid intermediates, further differentiating into short-lived immune and blood cell lineages (Orkin and Zon, 2008). SRPs play a vital role in immune defense, contributing to responses against infections, cancer, and radiation-induced immunosuppression (Ross et al., 2024; Hollingsworth et al., 2023). Among SRPs, long-term culture-initiating cells (LTC-ICs) are essential in restoring hematopoietic function after radiation exposure (Rodriguez-Fraticelli et al., 2018; Orkin and Zon, 2008). These cells can regenerate depleted precursor and progenitor populations, making them invaluable for treating patients exposed to high radiation doses (Biermann and Reya, 2022). However, despite their therapeutic potential, direct transplantation of SRPs presents significant challenges due to the difficulty in mobilizing and harvesting them in sufficient quantities for clinical application (Williams et al., 2024; Burt et al., 2008). Traditional methods, such as direct bone marrow aspiration from the iliac crest or *in vitro* expansion, are invasive and have logistical constraints. Meanwhile, cytokine-driven expansion requires prohibitively high doses, making it financially and biologically unfeasible. This underscores the need for novel strategies to enhance SRP availability, including gene therapy, combination treatments with cytokines and small molecules, and innovative approaches to preserving their self-renewal capacity while maintaining stemness.

This research topic combines cutting-edge methodologies and protocols that advance HSC proliferation and self-renewal. This research topic fosters critical discussions on optimizing SRP expansion and sustainability by consolidating insights from established and emerging techniques. As the field progresses, continued research will be essential to unlocking the full therapeutic potential of these self-renewing HSCs, paving the way for advancements in regenerative medicine and immune system restoration. The review by Cox et al. explored the role of Lin28b, an RNA-binding protein, in HSC development and function. Lin28b is predominantly expressed in fetal hematopoietic stem and progenitor cells (HSPCs) and is crucial in regulating the transition from fetal to adult HSCs. This review highlights how Lin28b influences HSC expansion and differentiation during early development, providing valuable insights into potential strategies for enhancing *ex vivo* HSC expansion. By leveraging the regulatory mechanisms controlled by Lin28b, this study offers promising avenues for improving HSC self-renewal and advancing therapeutic applications in regenerative medicine. Jia et al. investigated the effects of Polo-like kinase 1 (PLK1) inhibition on erythroid differentiation using both *in vitro* and *in vivo* models. PLK1 inhibitors, specifically GSK461364 and BI6727, significantly suppress erythroid cell proliferation. This suppression led to G2/M phase cell cycle arrest, increased apoptosis in erythroid cells, and the formation of abnormally nucleated late-stage erythroblasts. These findings underscore the critical role of PLK1 in erythroid differentiation and suggest that its inhibition could negatively impact normal erythropoiesis. This has potential implications for therapeutic strategies targeting PLK1, particularly in balancing anti-proliferative effects against possible hematopoietic side effects. Shin et al. examine the role of the key transcription factor TAL1 in erythropoiesis. TAL1 overexpression enhances red blood cell (RBC) production from induced pluripotent stem cells (iPSCs). TAL1 significantly improves hematopoietic differentiation, increasing the yield of glycophorin A^+^/CD71^+^ cells and gamma hemoglobin levels. However, its overexpression reduces enucleation efficiency, posing a challenge in generating mature, transfusable RBCs. Morino-Koga and Yokomizo reviewed key signaling pathways and mechanisms essential for HSC development. They discuss the transition of hemogenic endothelium to HSPCs, emphasizing the roles of specific signaling molecules and transcriptional regulators such as Wnt, Notch, and BMP in regulating HSC proliferation, differentiation, and self-renewal. This review highlights the importance of the microenvironment, particularly niche cell interactions, in maintaining the HSC function. Understanding these mechanisms is essential for improving hematopoiesis and blood-related disorders therapies. Kitajima et al. investigated the role of the transcription factor Lhx2 in the *ex vivo* expansion of HSPCs derived from human iPSCs. This study explores how modifying Lhx2 activity enhances the proliferation and self-renewal of HSPCs, which is crucial for improving hematopoietic cell therapy. The findings provide insights into optimizing iPSC-based HSPC production for clinical applications, emphasizing the potential of manipulating key transcription factors to enhance stem cell yield. Luanpitpong et al. examined the role of O-GlcNAcylation, a post-translational modification in hematopoiesis. Editing *OGT* and *OGA* genes in human iPSCs reveals how O-GlcNAcylation influences HSC function and differentiation. This study provides a deeper understanding of the metabolic regulation of HSCs and identifies potential targets for therapeutic intervention. Collectively, these articles contribute significantly to the current understanding of HSC biology. They offer novel insights into the molecular mechanisms that regulate HSC proliferation and self-renewal, paving the way for advancements in regenerative medicine and treatments for hematological diseases.
